# Functional ultrasound (fUS) detects mild cerebral alterations using canonical correlation analysis denoising and dynamic functional connectivity analysis

**DOI:** 10.1162/IMAG.a.128

**Published:** 2025-09-02

**Authors:** Flora Faure, Cindy Bokobza, David Guenoun, Juliette Van Steenwinckel, Pierre Gressens, Charlie Demené

**Affiliations:** Physics for Medicine Paris, Inserm, ESPCI Paris-PSL, CNRS, Paris, France; Université Paris Cité, Inserm, NeuroDiderot, Paris, France

**Keywords:** functional ultrasound imaging, perinatal systemic inflammation, canonical correlation analysis denoising, dynamic functional connectivity, neuroinflammation

## Abstract

Functional ultrasound (fUS) is a promising imaging method for evaluating brain function in animals and human neonates. fUS images local cerebral blood volume changes to map brain activity. One application of fUS imaging is the quantification of functional connectivity (FC), which characterizes the strength of the connections between functionally connected brain areas. fUS-FC enables characterization of important cerebral alterations in pathological animal models, with potential for translation into identification of biomarkers of neurodevelopmental disorders. However, the sensitivity of fUS to signal sources other than cerebral activity, such as motion artifacts, cardiac pulsatility, anesthesia (if present), and respiration, limits its capacity to distinguish milder cerebral alterations. Here, we show that using canonical correlation analysis (CCA) preprocessing and dynamic functional connectivity analysis, we can efficiently decouple noise signals from the fUS-FC signal. We use this method to characterize the effects of a mild perinatal inflammation on FC in mice. The inflammation mouse model showed lower occurrence of states of high FC between the cortex, hippocampus, thalamus, and cerebellum as compared with controls, while connectivity states limited either to intracortical connections or to ventral pathways were more often observed in the inflammation model. These important differences could not be distinguished using other preprocessing techniques that we compared, such as global signal regression, highlighting the advantage of canonical correlation analysis for preprocessing fUS data. CCA preprocessing is applicable to a wide variety of fUS imaging experimental situations, from anesthetized to awake animal studies, or for neonatal, perinatal, or neurodevelopmental imaging. Beyond fUS imaging, this method can also be applied to FC data from any neuroimaging modality when the sources of noise can be spatially identified.

## 1 Introduction

Functional ultrasound (fUS) is a promising imaging method for evaluating brain function and brain functional connectivity (FC) in both animal models and humans. fUS relies on measuring cerebral blood volume (CBV) changes linked to neuronal activity via neurovascular coupling (Deffieux et al., 2021). Since its introduction in 2011 (Macé et al., 2011), many studies have confirmed the correlation between fUS measurements and local neuronal activity (Aydin et al., 2020; Boido et al., 2019; Nunez-Elizalde et al., 2022). One application of fUS imaging is the assessment of resting-state FC in the brain (Bertolo et al., 2023; Hikishima et al., 2023; Osmanski et al., 2014). FC analyses are relevant for characterization of various animal models of various pathological conditions. For example, studies using fUS-FC have shown a decrease in FC in a double-hit rat model of perinatal brain injury (Mairesse et al., 2019), alterations of the sensorimotor network connectivity in rats with chronic arthritis (Rahal et al., 2020), and dynamic changes in brain connectivity following psychoactive substance administration (Rabut et al., 2020). fUS-FC has also been applied to human neonates in a study by Baranger et al. (2021), which showed reduced thalamo-cortical connectivity in preterm neonates as compared with term neonates.

In these prior studies, fUS was able to identify important changes in brain connectivity in severe pathological states. However, fUS’s capacity to assess milder alterations in FC is still at debate. Fluctuations in the measured fUS signal are primarily due to CBV changes related to cerebral activity, but several other sources may contaminate the fUS signal, including motion artifacts, cardiac pulsatility, level of anesthesia, and respiration. To ensure a robust but sensitive evaluation of brain connectivity with fUS imaging in diverse experimental settings, it is essential to establish reliable preprocessing and analysis methods for fUS imaging data that remove this contamination. This is crucial to retain only contributions that originate from brain activity, particularly in the context of milder cerebral alterations where subtle differences may be difficult to reveal when hidden by multiple noise sources.

**Fig. 1.**
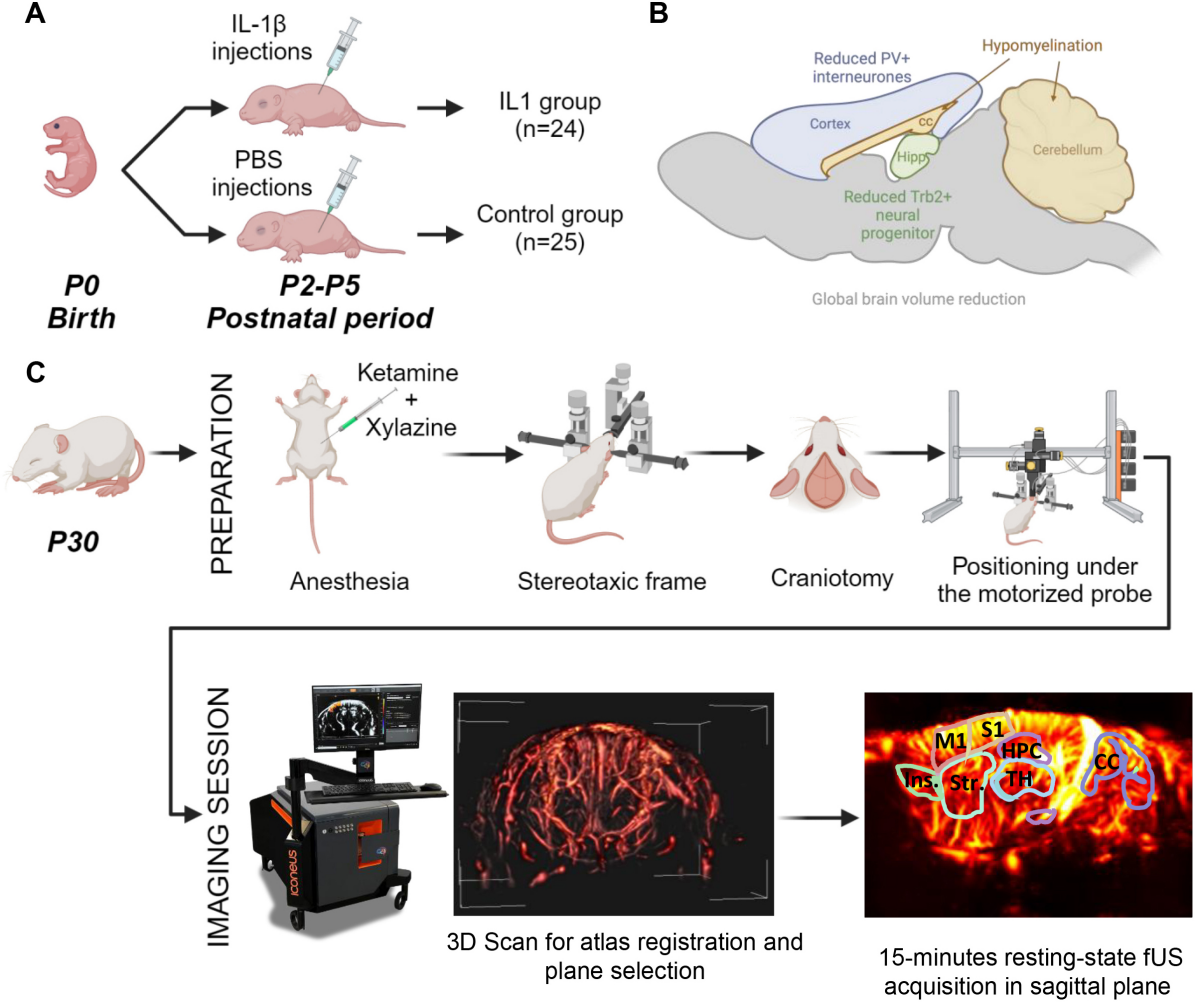
Protocol for assessment of changes in long-term functional connectivity using functional ultrasound imaging in a mouse model of IL-1β systemic perinatal inflammation. (A) Induction of the perinatal systemic inflammation model. Intra-peritoneal injection of Interleukin-1-beta (IL-1β) was performed between P2 and P5, twice per day. The control group received injection of saline (PBS). (B) Effects of the systemic perinatal inflammation include hypomyelination, a slight decrease of cerebral volume and impact on specific cell populations. (C) fUS imaging protocol at P30. Animal preparation (first row) includes ketamine–xylazine anesthesia, holding in a stereotaxic frame, craniotomy, and positioning. fUS imaging (second row) includes whole brain vascular imaging, then a 3D scan for functional atlas registration and selection of the acquisition plane that includes the functional areas of interest. Fifteen minutes of resting-state fUS imaging is then acquired at the selected parasagittal plane of interest.

In this study, we investigated the potential long-term effects of mild systemic perinatal inflammation on brain connectivity in mice using fUS imaging (Fig. 1A). Several prior studies have demonstrated a relationship between abnormal levels of inflammatory markers in the brain during the perinatal period and abnormal brain development, with resulting abnormal structural development, white matter injury, brain volume reduction, and later behavioral deficits (Cardoso et al., 2015; Favrais et al., 2011; Girard et al., 2012; Nist et al., 2020; Velloso et al., 2022). However, few studies have evaluated the relationship between inflammatory markers and functional brain activity (Morin et al., 2025) as this is methodologically challenging using traditional imaging methods such as fMRI or electrophysiology.

A systemic inflammation animal model can be developed using regular injections of low doses of interleukin-beta-1 (IL-1β), a molecule that mediates immune and inflammatory responses, in mice pups during the postnatal period (P1 to P5). This provoked inflammation induces structural and metabolic differences in the rodent brain (Fig. 1B), such as a reduction in myelination in the cingulum (Favrais et al., 2011), corpus callosum (Van Steenwinckel et al., 2019), and cerebellum (Klein et al., 2022). Inflammation also induces long-term changes in cortical parvalbumin-positive interneurons and their peri-neural nets (Stolp et al., 2019). Reduced Tbr2+ neural progenitor cell proliferation and microglial reactivity is also observed in the hippocampal dentate gyrus (Veerasammy et al., 2020), with a significant negative impact on memory (Favrais et al., 2011). These findings are also observed in human encephalopathy of prematurity (Volpe, 2009). Of note, the (IL-1β) inflammation model is relatively mild as compared with other models of inflammation, such as injection of lipopolysaccharide (LPS) (Favrais et al., 2022). As such, we expect that any differences in brain connectivity in the IL-1β model of inflammation would be less readily detectable than in an LPS model and chose to use this model to investigate whether fUS could detect the resultant subtle changes in brain FC. To detect the subtle connectivity differences induced by the IL-1β inflammation model, we use a new fUS pipeline analysis based on canonical correlation analysis (CCA) denoising and continuous dynamic functional connectivity (dFC) assessment. CCA is a tool used to describe common information (i.e., correlated) shared by two different datasets. In this study, we used CCA to characterize common noisy signals, including both physiological noise and motion artifacts, present in both functional and non-functional areas to remove them from the functional areas. CCA can be used to remove motion artifacts from fUS signals while maintaining a continuous time course, as compared with other preprocessing methods that typically suppress epochs with motion artifacts (Bertolo et al., 2023; Rabut et al., 2020). When performing a dFC analysis after CCA denoising to determine the succession of connectivity states, the preserved continuous time course allows for a more robust quantification of their occurrence rates, their mean dwell times, and the probabilities of transition between states.

Overall, we demonstrated that (1) CCA is an effective tool to remove contributions of noise sources from resting-state fUS signals and that (2) dFC analysis highlights changes in connectivity states occurrences, duration, and transitions in this mild inflammation (IL-1β) mouse model. This study shows that fUS imaging, with appropriate preprocessing and analysis pipeline, is sensitive enough to detect fine connectivity changes induced by systemic perinatal inflammation. Our findings pave the way for the characterization of mild cerebral alterations with fUS.

## 2 Methods

### 2.1 Animals and models

The protocol used in this study was approved by the Paris Nord ethics committee and the French Ministry of Research (APAFIS#18417-2018121914052786). A total of n = 49 male OF1 mice (Charles River, L’Arbresle, France) were used in this study and were imaged in 3 different experimental sessions (n = 15, n = 13 and n = 21, respectively). Mice from the same session were housed at the same time, with several weeks separating each session. Housing and experimental conditions were kept consistent across all animals. Mice received intraperitoneal (i.p.) injections twice a day from postnatal days P1 to P5. The IL-1β group (n = 24) received injections of 10 µg.kg-1 IL-1β (Miltenyi Biotec®) diluted in 5 µL of PBS (phosphate-buffered saline) 1X (n = 24). The control group (n = 25) received injections 5 µL of PBS 1X alone (control group n = 25) (Fig. 1A).

### 2.2 Functional ultrasound sequences

fUS acquisitions were performed using the IconeusOne ultrasound scanner (Iconeus, Paris, France) and the IcoPrime linear ultrasound probe (15 MHz, 128 elements, Iconeus, Paris, France) mounted on a 4-axis motor system. Ultrafast compound plane wave imaging data (Montaldo et al., 2009; Tanter & Fink, 2014) were acquired at plane wave repetition frequency of 10.5 kHz (11 compounded tilted plane waves ranging from -10° to 10° (2° steps), with a final compounded frame rate 500 Hz). Frame-to-frame complex correlation of these data enables local tissue velocity estimation (Loupas et al., 1995), and time integral of this velocity enables tissue motion estimation. Additionally, Singular Value Decomposition (SVD) clutter filtering (Demené et al., 2015) of blocks of 200 ultrafast frames was used to isolate the blood motion signal from surrounding tissue. The energy of the blood motion signal was computed to create Power Doppler images at a frequency of 2.5 Hz.

### 2.3 Functional ultrasound imaging sessions

On postnatal day P30, animals were prepared for the imaging session (Fig. 1C). Anesthesia was induced by intraperitoneal injection of a blend of xylazine (15 mg/kg) and ketamine (75 mg/kg). The anesthetized mice were placed on a stereotaxic frame for craniotomy and then placed under the imaging system. Each imaging session (Fig. 1C) began with an initial motorized anteroposterior 3D Power Doppler scan using the Iconeus software included in the ultrasound scanner. This vascular 3D-scan is registered to a vascular reference by the Iconeus software (Bertolo et al., 2021; Nouhoum et al., 2021). This vascular reference is linked to a functional MRI atlas (Allen Mouse Brain Common Coordinates Framework; Wang et al., 2020), enabling navigation through the imaged brain in real time. The probe is then placed in a sagittal position to select a plane of interest to image simultaneously the neocortex, the hippocampus, and the cerebellum. These regions were of particular interest as they have been previously shown to be affected during the perinatal period in IL-1β animal models of inflammation, with associated deficits in behavior (Favrais et al., 2011; Klein et al., 2022; Veerasammy et al., 2020). Each resting-state fUS acquisition was started 30 minutes after anesthesia induction to reduce variability regarding the depth of anesthesia across animals. fUS acquisition sessions lasted 15 minutes. During each acquisition, the temperature of the animal was monitored using an anal probe and maintained within a stable range using a thermoregulated heating mat. The animal’s respiratory rate was also monitored to ensure stable breathing during the imaging session.

### 2.4 Exclusion criteria

Animals were excluded from the analysis if (1) the craniotomy surgery induced any vascular anomalies such as hemorrhage or injury to a non-vascularized zone or (2) if the relative Doppler movie of the acquisition showed signs of cortical spreading depression (CSD). CSD is a wave of electrophysiological hyperactivity that propagates in the cortex that is followed by a wave of inhibition. CSD can be detected by Ultrafast Doppler with hyperemia followed by hypohemia and can be elicited by cranial surgery (Ayata & Lauritzen, 2015; Bourgeais-Rambur et al., 2022). Based on these criteria, a total of 13 animals were excluded (7 from control group and 6 from IL-1β group). An additional animal was excluded from further analysis as one of the regions of interest (ROIs) was not within the field of view.

### 2.5 Preprocessing of fUS data for functional connectivity assessment

For the reasons outlined above, appropriate preprocessing is crucial to remove any non-functional contributions to the fUS signal (proportional to the CBV). Indeed, the fUS signal can be corrupted by several noise sources, some being external to the animal and some being physiological or anesthesia induced. For instance, since fUS imaging is sensitive to motion (primarily of the red blood cells), vibration of the imaging setup or animal motion can corrupt the signal for a short period of time. Also, changes in the level of anesthesia and cerebral autoregulation can add slow drifting trends to the fUS signal. These noise sources possess very diverse spectral and temporal characteristics, making the use of simple approaches such as spectral filtering ineffective. Other widely used approaches, such as global signal regression (GSR) or independent component analysis (ICA)-based methods (Power et al., 2014), may introduce anticorrelation in connectivity analyses (Murphy & Fox, 2017) or a necessitate time-consuming manual screening of noise components (Griffanti et al., 2017). Other methods exist, for example, in fMRI noise removal using principal component analysis (PCA) based on the signal outside the brain (Chuang et al., 2019), and strategies have been developed to automatically detect noise components in ICA-based methods, such as ICA-AROMA (Pruim et al., 2015). Here, we present CCA denoising, which is applied before standard filtering of the functional frequency domain and normalization of the data.

Given two vector datasets, CCA seeks to determine a linear combination of vectors from each dataset, and build a set of pairs of vectors maximizing the correlation within the pairs, while also being otherwise orthogonal within the set. In other words, CCA finds a new basis for describing two sets of data while explaining the maximum correlation between them. In the context of this fUS study, the idea is to define two fUS datasets (Fig. 1A), $\;X_{f},\;$ a matrix of fUS signals from pixels in the functional area, and $X_{n}$, a matrix of fUS signals from pixels in the noise area (Fig. 1B), so that CCA can find a common basis to describe physiological noise and motion artifacts. This implementation is an extension of CCA denoising as described in Landemard et al. (2021).

**Fig. 2.**
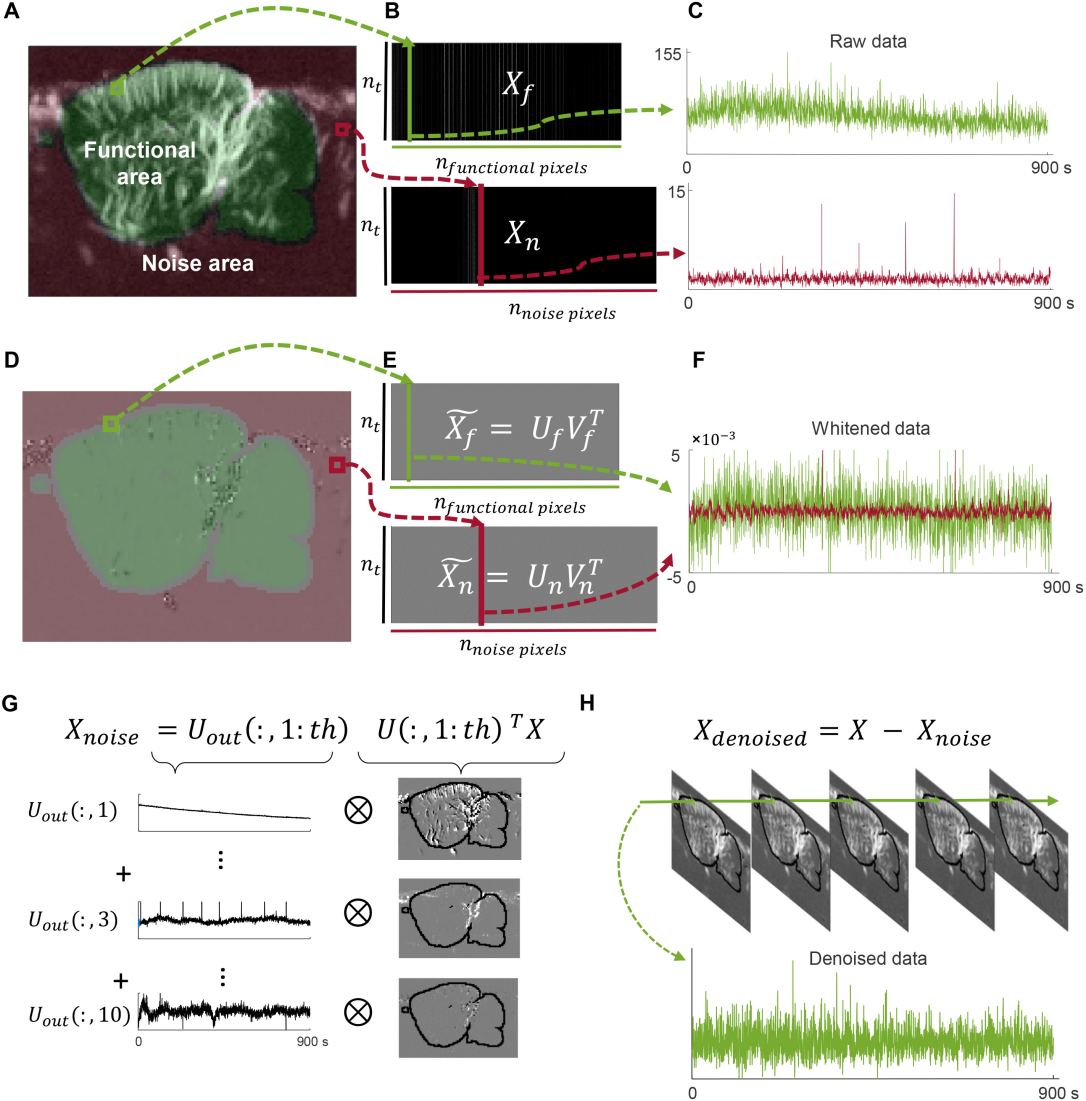
Canonical correlation analysis (CCA) denoising steps. (A) Spatial definition of two datasets: delimitation of the brain functional region of interest (ROI) (green) and the noise ROI (red) on the average fUS image. (B) Functional data and noisy data are arranged in two matrices $X_{f}$ and $X_{n}$ whose dimensions are number of time points x number of pixels (C). One column of those matrices is, therefore, the fUS signal time course in one pixel. (D) Data whitening. Effect of the data whitening of the spatial image (removal of relative amplitude information). (E) The whitened matrices ${\tilde{X}}_{f}$ and ${\tilde{X}}_{n}$ are used in the second step of the CCA (concatenation before singular value decomposition). (F) Pixel time courses after data whitening. (G) Projection on the common noise basis and threshold selection to determine the noisy signal to remove from the functional signal (green pixels). X is the concatenation of both datasets $[X_{f}\;X_{n}],$ U is the component of SVD decomposition of the concatenated whitened data $[{\tilde{X}}_{f}\;{\tilde{X}}_{n}]=USV^{T}$. (H) Denoising of the signal. The noise is removed from the original data in every pixel. Time course of the denoised signal of the selected functional pixel from functional area.

A popular implementation of CCA uses a change of basis for the datasets based on a whitening operation. This is because the CCA operation aims to maximize correlation (and not covariance) between the pairs of canonical vectors. This helps handle any signals that have very different amplitudes between the two datasets (Fig. 2C) by removing the amplitude information from every pixel (Fig. 2D, E) and keeping only information of temporal signal shape (Fig. 2F).

To perform signal whitening, each dataset is decomposed using SVD:

$$X_{f}=U_{f}S_{f}V_{f}^{T}\;and\;X_{n}=U_{n}S_{n}V_{n}^{T}.$$ (1)

And whitened matrices are computed from the singular vectors (Fig. 2E):

$${\tilde{X}}_{f}=U_{f}V_{f}^{T}\;and\;{\tilde{X}}_{n}=U_{n}V_{n}^{T}.$$ (2)

Then, SVD is applied on concatenated whitened datasets:

$$\left[{\tilde{X}}_{f}{\tilde{X}}_{n}\right]=USV^{T}.$$ (3)

U is the new common basis on which data can be projected to determine the canonical components that maximize correlation between datasets.

The first canonical components contain the common information between the two datasets. This common information should correspond only to physiological noises and motion artifacts, present both within and outside the brain, that we want to remove from the brain signal. The dimension of the noise subspace is defined through the choice of a threshold $th_{CCA}$ to reconstruct the noise signal (Fig. 2G). Then, we remove this noise signal from our signal of interest (Fig. 2H).

Selecting an adapted threshold is crucial to properly remove the noise signal. A threshold that is too low will result in residual noise in the data, while a threshold that is too high might remove meaningful functional information from the data. To select the appropriate threshold, we first computed, for varying $th_{CCA}$ values from 1 to 20, the correlation between the average denoised signal across all functional pixels and a signal quantifying the average tissue motion. Briefly, the quantification of tissue motion is calculated from the time-lag phase-correlation of the raw ultrasonic images, a method widely used for motion estimation in ultrasound imaging and elastography (Bercoff et al., 2004; Demené et al., 2021; Tanter & Fink, 2014). Then, we defined the minimal value for which the calculated correlations are significant at p < 0.001 (one-tailed). Applying the Fisher’s Z-transform, we want z > 3.1 (p < 0.001), corresponding to r > 0.1 for our sample size. Therefore, we selected the $th_{CCA}$ value for which the correlation averaged over all animals was lower than 0.1 to remove significant correlation with the signal from average tissue motion.

The CCA-denoised data are then filtered using a zero-phase 4th-order Butterworth bandpass filter in the [0.01 0.1] Hz frequency band and standardized.

### 2.6 CCA denoising efficiency

To quantify the effect of CCA denoising on correlation, we computed correlation matrices between pixels from functional area and noise areas before and after CCA denoising for each animal. We considered two sub-matrices: the functional-to-functional matrix, which includes only correlation coefficients between pixels within functional areas, and the functional-to-noise matrix, which includes correlation coefficients between pixels in functional and noise areas.

For each of this sub-matrix, we calculated the percentage of correlation values above a threshold of 0.1. These percentages were compared across the two sub-matrices and denoising conditions using ANOVA, followed by Tukey’s post hoc test.

For the seed-based maps, we used a spatial smoothing on the fUS dataset using a Gaussian filter with a full width at half maximum (FWHM) of 0.5 mm. A bandpass filter between 0.01 and 0.1 Hz was applied prior to computing the seed-based maps. All seeds were extracted from the registered atlas.

### 2.7 Dynamic functional connectivity (dFC) analysis

Compared to cruder approaches such as discarding epochs with motion artifacts (Bertolo et al., 2023; Rabut et al., 2020), using CCA denoising preserves a continuous time course in the fUS data. Moreover, these data present an appealing 0.4 s temporal resolution for dynamic functional connectivity analysis (dFC). We performed dFC analysis using a phase-based approach to describe the degree of synchronicity between functional regions of interests (ROIs) at every time point (Baranger et al., 2021; Rahal et al., 2020).

#### 2.7.1 Synchronicity matrices computation

**Fig. 3.**
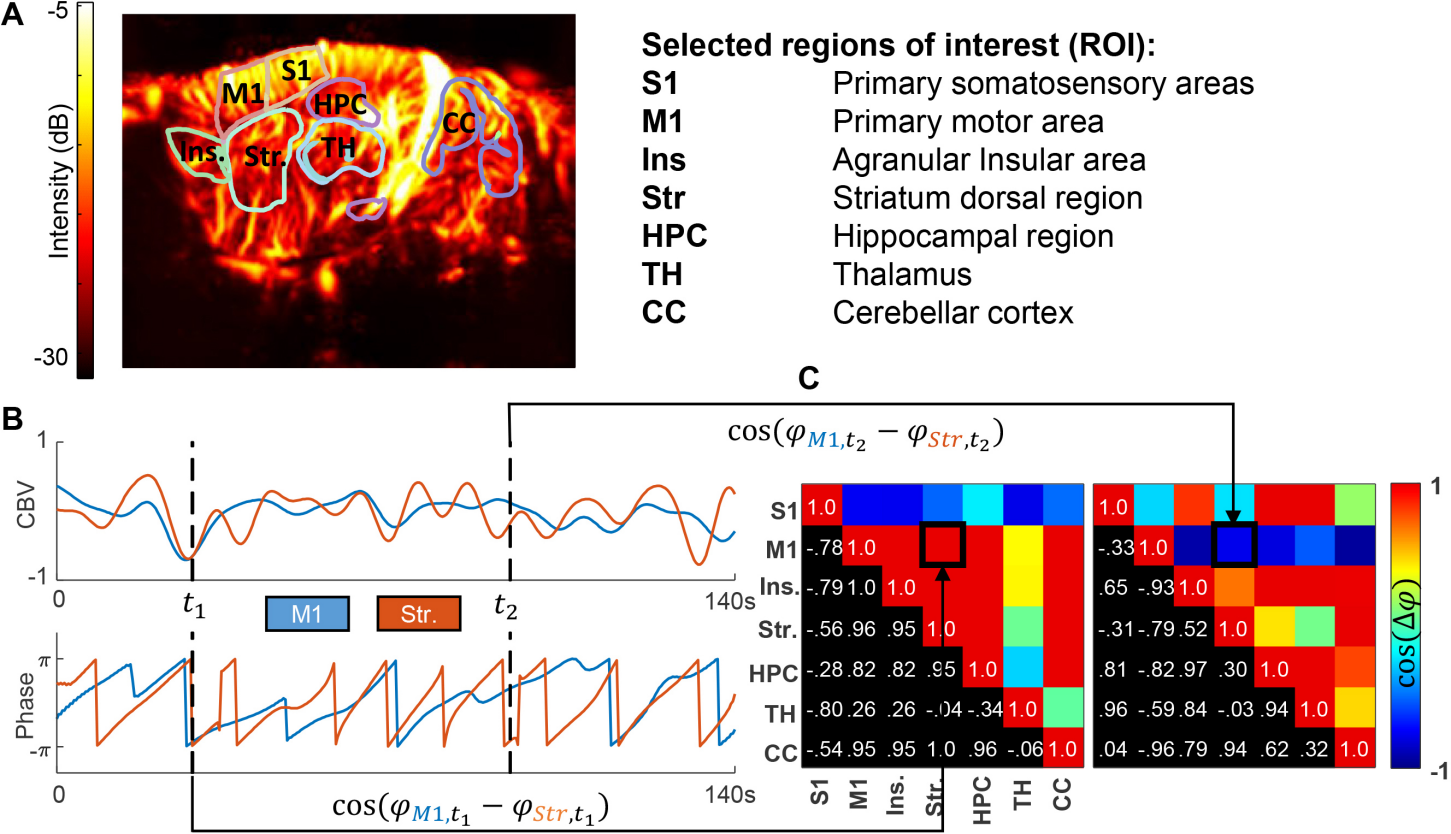
Synchronicity matrices construction. (A) Power Doppler image of the selected sagittal plane with delimitation of regions of interest (ROI) overlaid. (B) Computation of synchronicity matrices. Within ROIs (here the motor areas in blue and the striatum in orange), the phase signal is extracted using a Hilbert transformation of the ROI-averaged CBV signal. For each time point, a synchronicity matrix is calculated by computing the cosine of the phase difference between the two ROI-averaged CBV signal. (C) Two examples of synchronicity matrices are shown at times $t_{1}$ and $t_{2}.$

After registration of the fUS image to the Allen atlas, we defined seven selected ROIs: primary somatosensory areas (trunk, upper, and lower limbs) (S1), primary motor areas (M1), agranular insular area (Ins.), striatum (Str), hippocampus (HPC), thalamus (TH), and cerebellar cortex (CC) (Fig. 3A). We spatially averaged the signals over these seven ROIs for each acquisition and selected a 14-minute time window, excluding 30 s at the beginning and at the end of the acquisition.

First, we extracted the phase φ(t) of the averaged CBV signal for each ROI by taking the argument (MATLAB’s function *angle*) of the Hilbert transform of the signal (Fig. 3B). For each time point, we calculated the cosine of the phase difference between the signals of two ROIs to have information about the synchronicity between these two ROIs. Performing this pairwise calculation between ROIs allowed us to construct a synchronicity matrix for each time point t  (Fig. 3C), where its coefficient $c_{t}(i,j)$ of the synchronicity between $i^{th}$ ROI and $j^{th}$ ROI at time t is equal to

$$c_{t}(i,j)=\cos(\varphi_{i}(t)-\varphi_{j}(t)).$$ (4)

A coefficient of 1 means that signals are in-phase and synchronous, while a coefficient of 0 means they are out-of-phase. The synchronicity matrices show evolving patterns of synchronicity: for example, we see at t1 a desynchronization of the primary somatosensory areas (S1) and all other brain ROIs and at t2 a desynchronization of signals between the primary motor area (M1) and the rest of the brain ROIs (Fig. 3C). The matrices computation over the time course of all acquisitions resulted in a stack of 73648 synchronicity matrices.

#### 2.7.2 K-means algorithm and centroid synchronicity matrices

To describe the underlying common states of the stack of synchronicity matrices, we used the unsupervised K-means clustering approach using *kmeans* function in MATLAB with 100 iterations, 100 repetitions, the L1-norm distance, and K = 4 clusters. Iteration refers to the number of steps in the k-mean iterative process (centroid calculation -> group assignment). Repetition refers to the number of times the algorithm is initialized by selecting *K* random matrices from the dataset before starting the iterative process. The solution with the lowest sum of points-to-centroid distances over all repetitions is returned. The algorithm returns for each point its associated cluster and for each cluster a centroid, that is, a mean synchronicity matrix representative of the cluster.

We performed leave-one-out cross-validation (LOOCV) to ensure that the centroid states found were not specific to a given animal. For each training set, we measured the L1-norm distance between centroid states obtained with n-1 animals and centroid states obtained with the total n number of animals. The mean of this distance over each iteration is compared with the distance between centroid matrices as a scale reference. We also assigned each synchronicity matrix of the left-out animal to one of the LOOCV centroid states based on the shortest L1-norm distance. This resulting assignment was compared with the assignment obtained with the K-means clustering with *n* animals. We defined a classification score for each left-out animal as the percentage of correctly classified matrices.

To visually check the clustered data, we perform principal component analysis (PCA) of the stack of synchronicity matrices to determine the first three components that explained the most variance. We then can represent the matrices in a 3D space whose basis is the 3 PC with their corresponding affiliation to a given cluster.

#### 2.7.3 Fractional occupancies, mean dwell time, and states transitions

For each subject, we considered the sequence of membership of each cluster, as the time courses of each centroid states. From this, we defined multiple biomarkers of dFC for each centroid state:

- fractional occupancy (FO), the number of occurrences of the given state over the number of total time points
- mean dwell time (MDT), the average number of consecutive time points attributed to the state
- state transition probabilities (TP). The probability of transition from state i to state j is calculated as the ratio between the number of transitions from state i to state j and the number of transitions from state i to all other states (excluding transitions from i to i, that is, transitions from one state to itself).

### 2.8 Statistical analysis

Statistical analyses between the control group (n = 18) and the IL-1β group (n = 17) were performed using MATLAB.

We compared the fractional occupancies of each centroid states using a two-way independent t-test and considered the difference significant after Bonferroni correction with a degree of freedom equal to number of states –1.

We compared the mean dwell time of each centroid using the Mann–Whitney U test since the distribution is bounded in zero and we expected a skewed distribution. Differences were considered statistically significant after Bonferroni correction with a degree of freedom equal to the number of states.

We compared the state transitions using two-way independent t-test and considered the difference significant after Bonferroni correction with a degree of freedom equals to the number of transitions so (number of states –1) × number of states.

The results are presented as mean  ±  standard  deviation and with Bonferroni-corrected p-values.

### 2.9 Classification and ROC curves

We selected dFC biomarkers that were statistically significant and created a combination of these biomarkers using logistic regression model. We evaluated the classification abilities of this model using receiving operator characteristics (ROC) curves, for which we calculated the area under the curve (AUC) and the specificity and sensibility of the model based on the Youden index.

## 3 Results

### 3.1 CCA denoising is effective in removing noise components

**Fig. 4.**
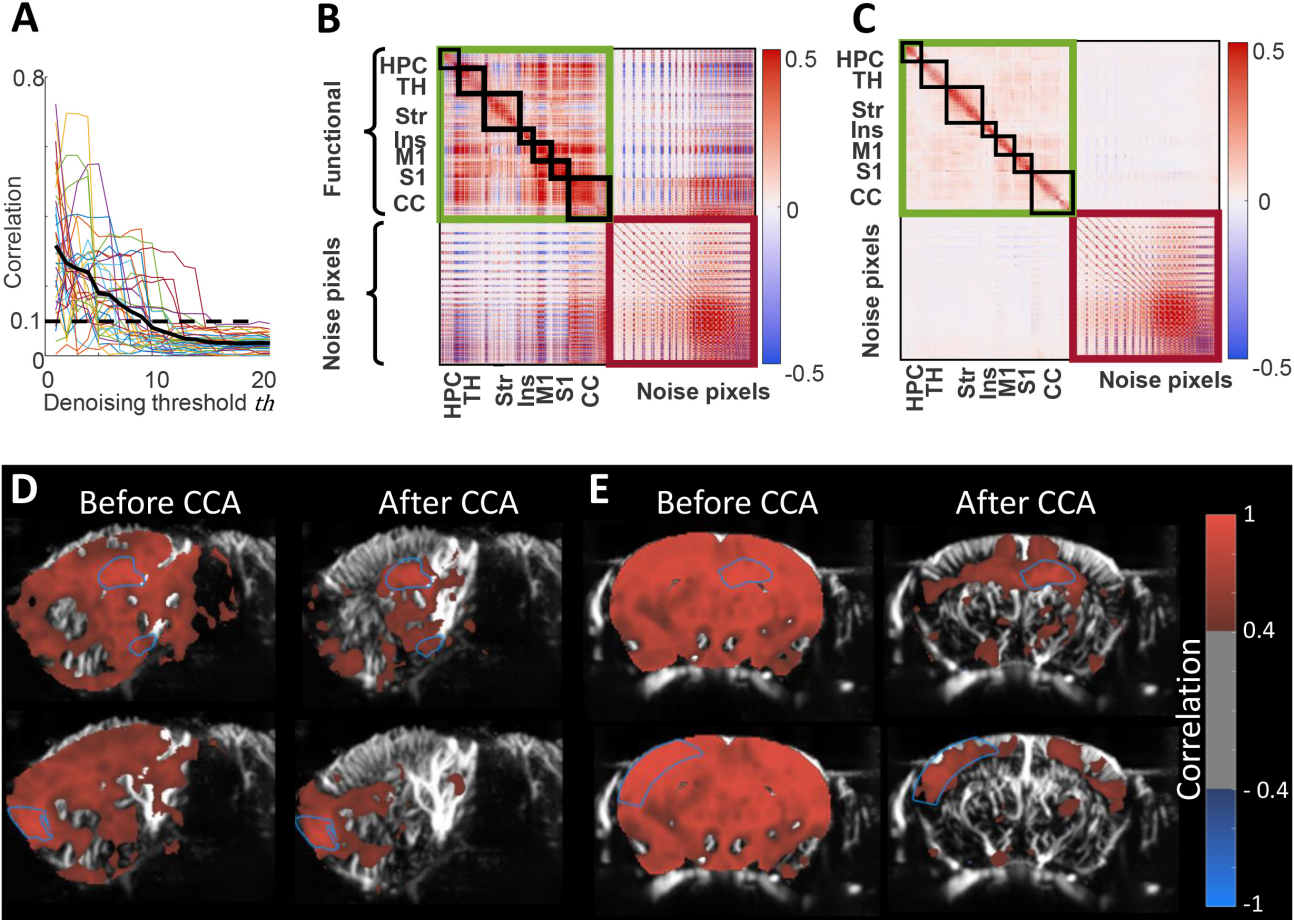
Canonical correlation analysis (CCA) denoising effects. (A) Threshold determination: Correlation between the averaged absolute tissue motion and the averaged denoised signal for varying denoising threshold values is plotted. Each colored line represents data from an individual mouse. The black line represents the mean correlation value across all mice. (B, C) CCA denoising modifies the correlation between pixels. Correlation matrices between pixels from functional and noise areas before CCA denoising (B) and after CCA denoising (C) are shown for a given mouse. (HPC = Hippocampus; TH = Thalamus; Str = Striatum; Ins = Agranular Insula; M1 = Motor cortex; S1 = Somatosensory cortex; CC = cerebellar cortex.). Green boxes highlight the “functional-to-functional” sub-matrix, red boxes highlight the “noise-to-noise” submatrix, with the functional-to-noise (symmetrical) sub-matrix being the upper right corner of the matrix. (D, E) CCA reduces the global level of correlation without concealing relevant functional connectivity, such as inter-hemispheric connectivity. (D) Seed-based correlation maps for a control mouse with a hippocampus seed (top) and an agranular insula seed (bottom) before (left) and after (right) CCA denoising. (E) Seed-based correlation maps for a control mouse presenting a high level of correlated noise with a hippocampus seed (top) and a cortical seed (bottom) before (left) and after (right) CCA denoising.

The minimum CCA threshold that ensured that the average correlation between absolute brain tissue motion and the denoised signal (black bold line in Fig. 4A) remained below the cutoff significant correlation values of 0.1 (p = 0.001, one-tailed t-test) was *$th_{CCA}=10$ .* We, therefore, applied this threshold of 10 for CCA denoising of all animals to remove noise components.

The impact of CCA denoising on the fUS signal is illustrated on an individual basis through the computation of the correlation between fUS signals before and after CCA (Fig. 4B, C). The correlation matrix between the pixel signals from distinct brain regions in a single animal prior to CCA denoising (Fig. 4B) shows a strong magnitude of correlation between functional and noise areas. Following CCA denoising (Fig. 4C), the same matrix reveals a notable decrease in the correlation between functional and noise regions. Quantitatively, on all animals, the average percentage of correlation coefficients above the threshold of 0.1 in the functional-to-noise submatrix is much lower after CCA than before (2 ± 1% vs. 35 ± 16%, p < 0.001). This demonstrates that the CCA denoising approach is efficient in removing contributions from signal fluctuations unrelated to brain activity. Also, within the functional area, the correlation submatrix exhibits a diminished correlation between pixels belonging to different brain regions. This is confirmed by a reduced average percentage of coefficients from 21 ± 5% to 7 ± 3% (p < 0.001). However, this percentage remains significantly higher than the functional-to-noise correlation level after CCA (7 ± 3% vs. 2 ± 1%, p < 0.05). Within the noise region, the correlation remains unchanged as the denoising process is only applied to pixels from functionals areas. This diminution of the global level of connectivity is not to the detriment of relevant FC, as shown in the seed-based analysis in sagittal (Fig. 4D) and coronal (Fig. 4E) imaging planes: for example, the coronal imaging plane still shows strong levels of inter-hemispheric connectivity (see also Supplementary Fig. S1 for more illustrative examples). The CCA-denoised fUS signals were then fed to the dFC analysis for synchronicity matrix clustering.

### 3.2 The four centroid connectivity states found with the K-means algorithm are robust and accurately describe individual brain state sequences

**Fig. 5.**
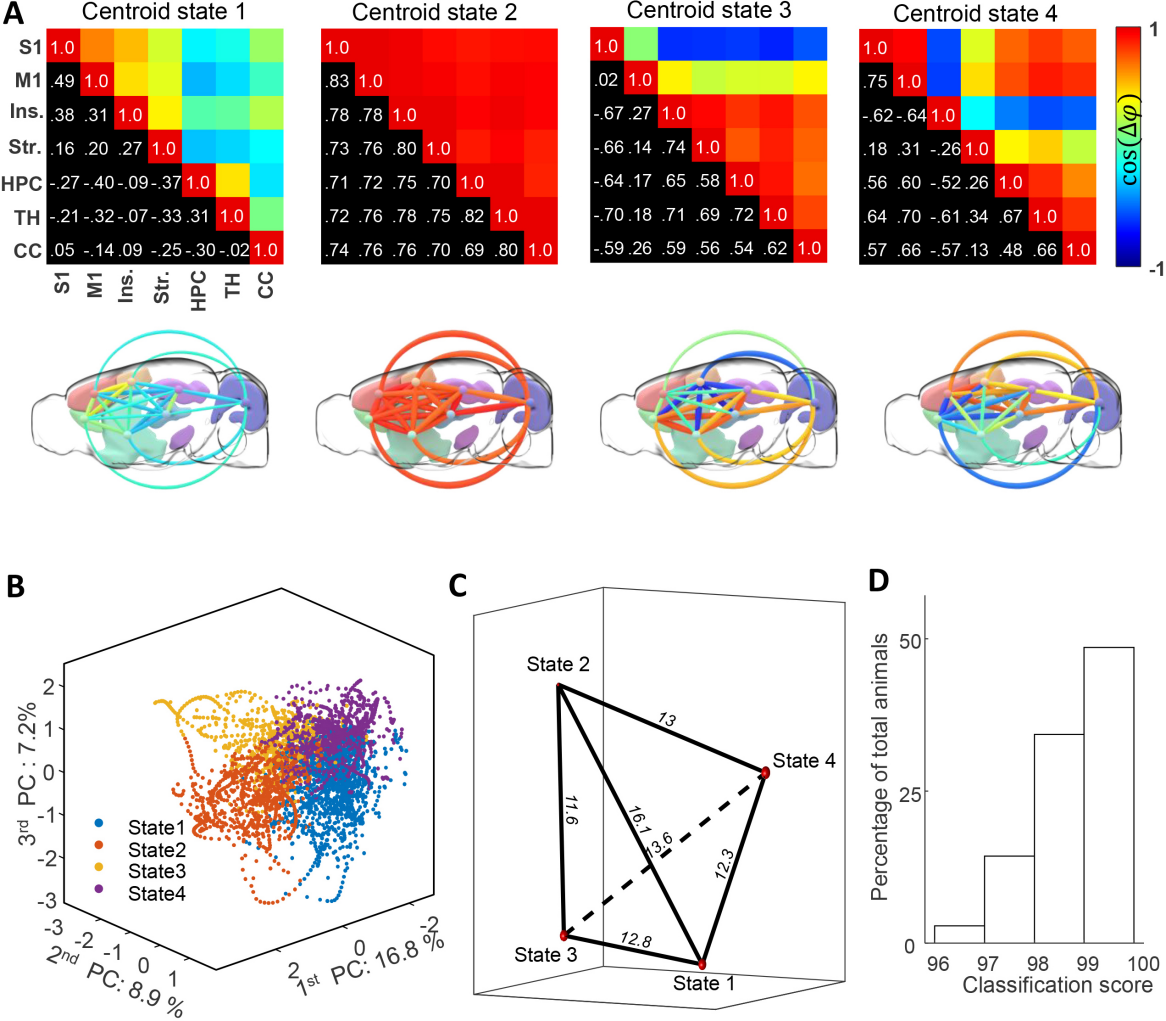
(A) Centroid states resulting from the K-means algorithm for K = 4. States are sorted by increasing total occurrences. Below each matrix, a 3D representation of the brain state, with links representing the corresponding coefficients of the matrix. The same colormap is used for matrix and 3D representations. (B) Representation of the clustered synchronicity matrices for one animal. Individual synchronicity matrices are represented by points in a 3D space whose basis is the first three principal components of the principal component analysis performed on all matrices. Each color shows the corresponding affiliation to cluster determined by the K-means algorithm. (C) Validation of states robustness with leave-one-out cross-validation (LOOCV). States are represented in a 3D graph so that the edges (black lines) are proportional to the L1-norm distances between the matrices. At each node, the radius of the red sphere is the averaged distance between the states found with N mice and the states found with N-1 mice at each iteration of the LOOCV. (D) Validation of states classification. At each LOOCV iteration, we compute a classification score for the left-out animals as the number of time points correctly classified divided by the total number of time points. The histogram shows the percentage of animals within each band score.

The four centroid states returned by the K-means algorithm, which was applied to the stack of synchronicity matrices from all acquisitions, are presented in Figure 5A. They are represented in the form of synchronicity matrices (top) and connectome maps (bottom), and sorted based on their total occurrence across all animals:

- Centroid state 1: a state of cortico-cortical synchronicity with desynchronization with the rest of the brain + hippocampus and thalamus synchronicity
- Centroid state 2: state of global synchronization between all structures within the hemisphere
- Centroid state 3: state of desynchronization of motor and sensory areas with the rest of the hemisphere
- Centroid state 4: state of desynchronization of agranular insula and striatum with the rest of the hemisphere

The trajectory across these centroid states during an imaging session is shown for one animal in Figure 5B. The synchronicity matrices are represented as positions in a 3D space, whose bases are the first three PCs from the PCA of the whole matrix dataset. Each cluster assignment is displayed as a different color. It should be noted that even if the three PCs explain only 32.9% of the total variance, the boundaries of the four states are clearly defined in this 3D subspace. The visible trajectories of points that represent the temporal sequence of matrices allow for clear visualization of state transitions.

The LOOCV results for the K-means algorithm demonstrate that these four states are both robust and not specific to a single animal. At each iteration, we calculated the L1-distance between states found with N-1 animals and their corresponding states found with N animals. The averaged distance for each state is relatively small $(d_{state\;1}\;\;=\;\;0.23\;\;\pm \;\;0.10,\;d_{state\;2}=\;\;0.07\;\;\pm \;\;0.04,\;d_{state\;3}\;\;=$ $0.23\;\;\pm \;\;0.23\;\;\pm \;\;d_{state\;4}\;\;=\;\;0.26\;\;\pm \;\;0.14$ ) in comparison with the mean intercluster distance, which is used as a reference distance ($d_{inter}\;\;=\;\;13.23\;\;\pm \;\;1.56)$ (Fig. 5C). Moreover, the LOOCV demonstrates that these states are capable of accurately identifying the sequence of states for a novel animal. The classification score calculated for each left-out animal is consistently above 96%, with 50% of the animals having a score above 99% (Fig. 5D).

### 3.3 The inflammation group has a different pattern of dFC that shows a decrease in connectivity

**Fig. 6.**
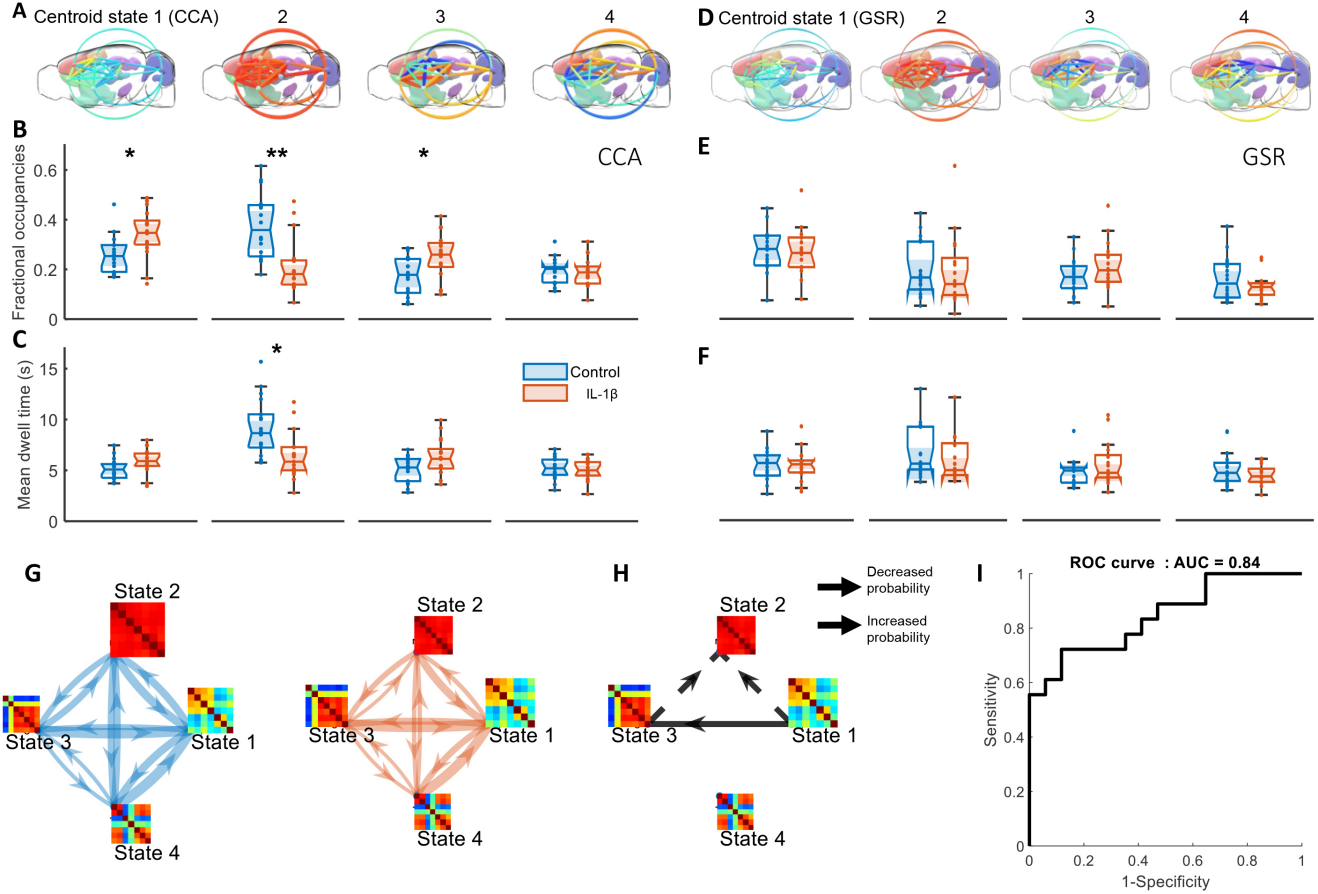
IL-1β mice have a different pattern of dynamic functional connectivity (dFC), as revealed by CCA preprocessing. (A) Brain states after CCA denoising. 3D representation of the brain state as introduced in Figure 5, when using CCA denoising as a preprocessing. (B) Fractional occupancies (CCA denoising). Two-way t-tests were performed between the control group (blue) and the IL-1β group (orange) for each centroid state, followed by Bonferroni correction for multiple comparisons with a degree of freedom equal to the number of states –1 (i.e., 3) (*p < 0.05, **p < 0.01, ***p < 0.001). (C) Mean dwell times (CCA denoising). Mann–Whitney U test was performed between the control group (blue) and the IL-1β group (orange) for each centroid state, followed by Bonferroni correction for multiple comparisons with a degree of freedom equal to the number of states (i.e., 4) (*p < 0.05, **p < 0.01, ***p < 0.001). (D) Brain states after global signal regression (GSR) preprocessing. 3D representation of the brain state when using GSR as a preprocessing. (E) Fractional occupancies (GSR). Two-way t-test analysis was performed between the control group (blue) and the IL-1β group (orange) for each centroid state, followed by Bonferroni correction for multiple comparisons, showing no significant differences across states. (F) Mean dwell times (GSR). Mann–Whitney U test was performed between the control group (blue) and the IL-1β group (orange) for each centroid state, followed by Bonferroni correction, showing no significant differences across states. (G) State transitions for the control group (blue arrows) and the IL-1β group (orange arrows) after CCA denoising. The width of the arrows is proportional to the probability of transition from one state to another. The size of the matrices is proportional to their fractional occupancies. (H) Significant differences in state transition (CCA denoising). Two-way t-test was performed followed by Bonferroni correction for multiple comparisons with a degree of freedom equal to the number of transitions (i.e., 12). Dashed arrows represent a significantly decreased probability of transition, and the full arrow a significantly increased probability of transition (p < 0.05). (I) Classification evaluation performance of dFC biomarkers (CCA denoising). Receiving operator characteristics (ROC) curve obtained for the logistic regression model of the following dFC biomarkers: Fractional occupancies of states 1, 2, and 3 and mean dwell time of state 2. AUC = area under the curve.

Comparison of the control group and the IL-1β group for the three selected dFC biomarkers (fractional occupancies (FO), mean dwell time (MDT), and transition probabilities (TP)) reveals a modified pattern of dFC with reduced connectivity for the IL-1β group (Fig. 6). This result remained the same when performing the K-means algorithm with K = 3 or K = 5 states (Supplementary Fig. S1).

The reduced connectivity is shown by a significant decrease of FO for global synchronicity state 2 in the IL-1β group ($FO_{control}=0.37\;\;\pm \;\;0.13,\;FO_{\text{IL}-1\beta }=0.21\;\;\pm \;\;0.11,\;\;p=$  0.001 ), accompanied by a significant increase of FO for states of lower global synchronization 1 ($FO_{control}=0.26\;\;\pm \;\;$ $0.08,\;FO_{\text{IL}-1\beta }=0.35\;\;\pm \;\;0.10,\;p=0.018$ ) and 3 ($FO_{control}=$ $0.17\;\;\pm \;\;0.08,\;FO_{\text{IL}-1\beta }=0.25\;\;\pm \;\;0.09,\;p=0.018$ ) (Fig. 6A, B).

The reduced FO of state 2 can be partially explained by the MDT of state 2 which is significantly shorter in the IL-1β group ($MDT_{control}=9.14s\;\;\pm \;\;2.80s,\;MDT_{\text{IL}-1\beta }=6.39s\;\;\pm \;\;$ 2.31s, p=0.012 ) (Fig. 6C).

However, in the absence of CCA denoising, and despite very similar brain states (Fig. 6D) (Supplementary Fig. S3), significant differences in the dFC pattern were not observed between the two groups for FO (Fig. 6E) or MDT (Fig. 6F) when GSR and suppression of noisy epochs were used to denoise the signal.

Back to the case of CCA denoising, the analysis of state transition probabilities (TP) gives complementary information on the states’ fractional occupancies. For each group, we can represent the TP as a directed graph (Fig. 6G) (see Supplementary Fig. S3 for comparison with GSR). The arrow head indicates the state to which the brain transitions, and the width of the arrows is proportional to the probability of transition. We observed a decreased probability of transition in the IL-1β group from state 1 to state 2 ($TP_{control}=0.36\;\;\pm \;\;0.10,\;TP_{\text{IL}-1\beta }=0.25\;\;\pm \;\;0.08,\;p=0.019$ ) and from state 3 to state 2 ($TP_{control}=0.32\;\;\pm \;\;0.08,\;TP_{\text{IL}-1\beta }=0.21\;\;\pm$ 0.10  p=0.012 ) (dotted arrow Fig. 6H). This also explains partially the reduced fractional occupancy of state 2. We also observed an increased probability of transition from state 1 to state 3 ($TP_{control}\;\;=\;\;0.27\;\;\pm \;\;0.10,\;TP_{\text{IL}-1\beta }\;\;=\;\;0.38\;\;\pm \;\;0.09,$ p=0.011) (full arrow Fig. 6H) that could explain the increased occurrence of state 3.

### 3.4 dFC biomarkers successfully classify animals with inflammation

The logistic regression model using FO of states 1, 2, and 3 and MDT of state 2 is significantly different of a constant model (p < 0.01). The ROC curve obtained from this model (Fig. 6I) gives an AUC of 0.84 with a specificity of 88% and sensitivity of 72%.

## 4 Discussion

In this study, we showed that CCA-based denoising of the fUS imaging signal can enable the detection of long-term modifications in dynamic functional connectivity (dFC) patterns induced by a mild systemic inflammation during the perinatal period in a preclinical model.

The model selected for this study was based on human clinical data from preterm infants. The neurodevelopmental outcomes of premature birth on brain development can vary widely, ranging from normal development to severe neurological deficits. The risk of major disabilities (cognitive impairment and/or cerebral motor deficits) increases with lower gestational age at birth and birth weight (Van Steenwinckel et al., 2024). A large proportion of infants born with extremely low birth weight develop a white-matter brain injury known as encephalopathy of prematurity (EoP) (Van Steenwinckel et al., 2024). Additionally, gray matter lesions, specifically impacting interneurons, have been reported in cohorts of preterm infants (Stolp et al., 2019). Prematurity has multifactorial etiologies, but chorioamnionitis—defined as acute inflammation of the amnion and chorion—remains the primary factor associated with preterm birth. More than 40% of infants born spontaneously before 32 weeks of gestation are exposed to inflammatory products via the chorion (Stolp et al., 2019). Studies in both humans and rodents have demonstrated a strong association between exposure to maternal inflammation during the neonatal period and later brain injury and neurodevelopmental disorders (NDDs). Numerous neurodevelopmental processes occur during the period corresponding to preterm birth (from 24 to 37 weeks of gestation), including neuronal migration, dendritic arborization, synapse formation, and glial cell proliferation and differentiation. Any disruption of these processes by intrinsic or extrinsic factors, such as inflammation, increases the risk of NDDs (Hagberg et al., 2015). In this study, we used a mouse model of perinatal mild systemic inflammation induced by repetitive injections of interleukin-1β (IL-1β) from postnatal day 1 (P1) to postnatal day 5 (P5). This developmental period corresponds to the last trimester of brain development in humans. This approach mimics neonatal inflammatory syndrome and replicates the brain injuries observed in recent preterm infant cohorts (Larroque et al., 2003; Marret et al., 2013; Verney et al., 2012). IL-1β-exposed mice exhibit increased microglial reactivity and diffuse WM lesions (Van Steenwinckel et al., 2019), similar injuries to those seen in preterm infants. A hallmark of the IL-1β model is the onset of oligopathy, characterized by impaired oligodendrocyte maturation without affecting proliferation or survival, along with persistent myelination defects (Favrais et al., 2011). Structural MRI analysis has revealed that systemic IL-1β treatment induces reductions in both gray and white matter volumes (Klein et al., 2022). Additionally, these animals show a reduction in parvalbumin-expressing interneurons, similar to what is observed in human preterm infants (Stolp et al., 2019). Behavioral analyses of IL-1β-exposed animals reveal various cognitive deficits, including memory impairment, anxiety-like behaviors, and social interaction deficits, but without motor disabilities (Bokobza et al., 2022; Favrais et al., 2011; Van Steenwinckel et al., 2019; Veerasammy et al., 2020). These same behavioral findings are also observed in human preterm infants (Agut et al., 2020).

In this study, we explored the use of canonical correlation analysis (CCA) denoising in fUS signal preprocessing to evaluate FC in IL-1β animal models. Our method offers the advantage of allowing for a continuous fUS signal, as compared with traditional preprocessing methods which suppress corrupted epochs, thereby biasing downstream FC analyses. Two steps are decisive in the implementation of CCA: (1) the selection of the noise area and (2) the choice of the CCA threshold. A cutoff threshold that is too low will not sufficiently remove noisy components. The selection of the noise area will define which noise sources can effectively be described by CCA and removed. For example, in our dataset, using a threshold of $th_{CCA}$ = 3 was insufficient to remove noise, and as a result, the FC metrics did not reveal any significant differences between groups (Supplementary Fig. S4). To determine which threshold to use, previous work has assessed the correlation between video-captured animal motion and the output signal (Landemard et al., 2021). However, external measurements of motion such as video are not always available. Ultrasound imaging presents the intrinsic advantage of being very sensitive to tissue motion, with a long history of method development for efficient tissue estimation (Bercoff et al., 2004; Demené et al., 2021; Tanter & Fink, 2014). Correlating the denoised fUS signal with this independent ultrasound-based tissue motion estimation helped us select a fixed threshold of 10 used for all animals that could be used to achieve effective denoising on an individual animal basis. Effective denoising was also observed for thresholds near this value (th = 8 and th = 12), with similarly significant differences in FC metrics between groups (Supplementary Fig. S4). This indicates that the CCA denoising method yields robust results and is not overly sensitive to the exact threshold, provided it is sufficiently high to remove noisy components. Moreover, as the implementation of CCA denoising relies on SVD, adaptive threshold selection techniques used for tissue motion filtering (Baranger et al., 2018) could be explored to optimize threshold selection. In a closely related method (Chuang et al., 2019), the authors estimated the first 10 principal components (PCs) of the signal outside the brain and scored which PCs were relevant to the signal inside the brain. Then, they used these relevant PCs as regressors for denoising the signal inside the brain. The advantage of our approach is that it leverages a signal decomposition intrinsically relevant to the data inside the brain, eliminating the need for sorting between PCs and limiting the risk of damaging the signals. We did not compare our method with an ICA-based denoising method, as we did not want to introduce prior knowledge of the temporal and spatial features and sort for the relevant components as done in ICA-AROMA (Griffanti et al., 2014; Pruim et al., 2015). We also showed that CCA is more sensitive than GSR, allowing us to detect significant differences in FC metrics between the control and IL-1β group with a larger effect size. The advantage of CCA denoising over GSR preprocessing in the case of our inflammation model is obvious: since one of the effects of inflammation is a reduction in global functional connectivity between the targeted areas, this effect will be greatly attenuated by a preprocessing method such as GSR which decreases the magnitude of general correlation. Since CCA finds signal correlations specifically from outside of the brain, the confounding effect of physiologically induced CBV variations can be disentangled from CBV variations induced by a genuine brain state of global connectivity.

We also studied dFC by using a phase-based approach to evaluate synchronicity matrices and extracted dFC biomarkers from the analysis of the clustered matrices. Previous fUS imaging studies have demonstrated altered patterns of dFC with this approach both in rodents and in human neonates under different pathological conditions (Baranger et al., 2021; Rahal et al., 2020). These studies involved very strong modifications of the connectivity and behavior, including chronic pain induction, scopolamine injection, and important differences in neurodevelopmental stage. Moreover, they focused only on comparing fractional occupancies of brain states. Here, we aimed to use an experimental condition that would induce mild differences between the control and experimental groups to assess whether the fUS dFC analyses could still detect differences between groups. Using the CCA denoising method, we were able to preserve the temporal continuity of the fUS signals. Therefore, we were also able to identify other dFC biomarkers such as mean dwell times and states transition probabilities. An important finding is that we could discriminate between this mild inflammation model and the controls using our fUS connectivity assessment, with potential for biomarker identification. This distinction could not be identified using classical preprocessing steps such as GSR. Some of the dFC differences between the control and IL-1β groups revealed in this article could have been also characterized using static connectivity: static connectivity revealed in the IL-1β group a significantly decreased connectivity nodal strength (Chiarelli et al., 2021) for the hippocampus (p < 0.01) and cerebellum (p = 0.05), in line with the increased occurrence of state 1 showing an asynchrony of those areas. But as compared with dFC analysis, static connectivity only gives one connectivity (correlation) matrix for each animal that collapses connectivity across the entire imaging session to a singular value, thereby obscuring important differences in state transition. While static connectivity can capture some of the effects of pathology on brain FC in animal models, it will inherently present a coarser granularity and possibly lower sensitivity because it is in a space of lower dimensionality.

A typical limitation of the K-means algorithm to cluster the matrices is the prior selection of the number *K* of representative states. We aimed to optimize the K-means algorithm to identify the global dynamic connections patterns that were common to all animals, and so we assumed that the required number of states *K* would be low. In this study, we defined four states and subsequently validated their robustness and accuracy in describing dFC across all animals, using LOOCV. We also showed that the observed differences in connectivity between the IL-1β and control groups were similar in the case of K = 3 and K = 5 (Supplementary Fig. S2).

The analysis of the dFC biomarkers showed that animals from the IL-1β group were less likely to transition in a state of global intrahemispheric synchronicity (state 2). When these animals were in State 2, they spent less time in this state as compared with the control group, resulting in a reduced occurrence of this state. Overall, these modifications of dFC biomarkers showed a decreased connectivity for the animals from the inflammation group. To date, few studies have focused on measuring FC in models of perinatal systemic inflammation to compare our results with, but they are in line with a study in which systemic inflammation by IL-1β injection was combined with fetal growth restriction and in which fUS imaging showed a reduction in inter- and intra-hemispheric connectivity, impaired behavior, and cognitive abilities (Mairesse et al., 2019). The myelination defects observed in this model of inflammation could potentially explain this reduced connectivity (Favrais et al., 2011). Other studies using acute postnatal neuroinflammation models—such as lipopolysaccharide (LPS) injection (mimicking bacterial infection) (Guevara et al., 2017) or viral infection by Zika virus (Mavigner et al., 2018)—also show reductions in connectivity. In humans, Rudolph et al. (2018) have shown a link between reduced connectivity and increased markers of maternal inflammation.

Moreover, recent findings support the hypothesis that neuroinflammation plays a role in the development and manifestation of ASD (Bokobza et al., 2019; Matta et al., 2019). Since the IL-1β model of inflammation we used in our study shows behavioral modifications such as anxiety-like behaviors and social interaction deficits without motor disabilities, similar to what is observed in ASD, our findings may in part explain these features seen in ASD. Studies have shown inconsistent results with both hyper- and hypo-connectivity of large-scale networks. However, a literature review by Uddin et al. (2013) highlighted a pattern with neurodevelopment: brain hyper-connectivity patterns are more seen in children, whereas brain hypo-connectivity patterns are prevalent in adolescents and adults. Since we evaluated connectivity at P30, a transitional stage between childhood and adolescence in the mouse, our findings of decreased global connectivity could be consistent with the hypo-connectivity observed in adolescents humans. In addition, the dFC biomarkers analysis showed an increased occurrence of state 1 and state 3 in the IL-1β group. The first state depicts a subcortical–cortical desynchronization: the thalamus and the hippocampus are synchronized but not with cortical areas and the striatum, while cortical areas are synchronized with each other and with the striatum. The increased occurrence of this state in our inflammation model also appears consistent with the findings of Di Martino et al. (2011), who reported increased FC in the striatum with cortical areas in children with ASD. The third state represents a desynchronization of the primary motor and sensory areas with the remaining brain regions of interest. This state appears to involve the sensorimotor network, which has been also shown to be impacted in ASD (Iannone & De Marco García, 2021; Oldehinkel et al., 2019). However, this state must be interpreted with care as the imaging plane is limited to one hemisphere. It is possible that both M1 and S1 areas are highly correlated to their contralateral areas during this state. Additionally, the dFC biomarker analysis did not show any differences for the fourth state which represents a desynchronization between the striatum, the insula, and the rest of the brain. The interpretation of this state is less evident and nothing similar has been shown in the literature to the best of our knowledge. However, the anterior insula is known to be part of the salience network (Uddin, 2015), which is involved in switching between brain states, particularly between the default mode network and the central executive network. The salience network also includes the dorsal anterior cingulate cortex (dACC), which is not visible in our imaging plane. Similarly to state 3, one hypothesis is that what we observe as a desynchronization of the insula in state 4 might actually represent a state in which the insula is highly synchronized with a hidden brain region, such as the dACC. Both the insula and striatum are involved in multiple sensory and cognitive processes and so state 4 might be a mix of states where they participate in distinct networks for which brain areas are not visible in the selected imaging plane. Overall, integrating dFC biomarkers showing significant differences between groups in a logistic regression model, we were able to classify animals between the control group and the IL-1β group with a strong degree of accuracy.

A limitation of the present study is that the evaluation of connectivity was confined to a single 2D slice and in a single hemisphere, thereby preventing to evaluate the interhemispheric connectivity and out-of-plane networks. It is established that interhemispheric connections and functional networks develop according to a spatio-temporal pattern (Smyser et al., 2010), which can be disrupted by neuroinflammation. The monitoring of these networks may facilitate the development of more accurate biomarkers from dFC analysis. The limitation of 2D imaging may be overcome in future studies with the recent development of matrix or multi-plane ultrasound probes for fUS imaging, enabling whole-brain 3D imaging of the mouse brain (Bertolo et al., 2023). Another limitation is that we used a young adult parcellation atlas reference (Allen Atlas; Wang et al., 2020), while the mice in our study were of adolescent age (P30). In future studies, we will implement a P30 reference atlas (vascular and parcellation) as new developmental templates have very recently become available (Kronman et al., 2024).

Our proposed CCA denoising method will very likely translate easily to the 2.5D (multi-slice; Bertolo et al., 2023) or 3D (matrix array) fUS imaging case, possibly even with improved efficacy as the non-functional area will capture a larger diversity of noise sources. As of now, these imaging strategies are costly (>60 k€ add on for multiplane) or may not be standardly available. Here our choice of 2D imaging was driven by the coplanarity of the targeted structures (cingulum, corpus callosum, cerebellum, and motor and somato-sensory cortex; Van Steenwinckel et al., 2019), along with ease of implementation.

We performed our fUS imaging acquisitions under anesthesia, which is known to significantly alter both static (Ferrier et al., 2020) and dFC quantifications (Gutierrez-Barragan et al., 2022). Nonetheless, we used anesthesia in our animal model for several reasons. First, because awake animal imaging is challenging to implement. Even if solutions exist for fUS imaging in awake animals (Bertolo et al., 2023; Tiran et al., 2017), most neuroimaging studies in rodents are currently performed under anesthesia. Second, our goal was to develop a processing pipeline able to provide robust biomarkers, even in the case of greater brain state variability such as changes in level of anesthesia, making it a potential preprocessing tool for the great majority of users. Moreover, ketamine–xylazine anesthesia has been shown to enable resting-state functional connectivity assessment, such as in Xie et al. (2020) via functional optical intrinsic signal imaging in C57BL/6J mice, comparable with what is observed in the literature in awake mice. We are confident that the presented CCA preprocessing for connectivity analysis will be an asset both for awake animal fUS imaging and neonatal fUS imaging, which we will confirm in future research.

Numerous clinical and preclinical studies have demonstrated a link between postnatal systemic inflammation, abnormal structural brain development, and behavioral and cognitive changes in childhood (Jiang et al., 2018; Malaeb et al., 2014). However, the precise mechanisms involved remain unknown, which limits the ability to accurately predict long-term neurodevelopmental outcomes for patients. fUS imaging has the potential to address this gap by assessing resting-state dFC in models of encephalopathy of prematurity in preclinical studies as well as in human neonates.

In conclusion, this article describes CCA denoising as a new preprocessing technique for efficient denoising of fUS imaging for dFC analysis. We have shown here that it can reveal long-term FC changes in a model of mild perinatal systemic inflammation. This illustrates the potential of fUS imaging as a tool for better understanding and monitoring of the onset of NDDs.

## Supplementary Material

Supplementary Material

## Data Availability

Code and example data supporting the CCA and dFC implementations are openly available in the Zenodo repository at https://doi.org/10.5281/zenodo.16893295. Raw data may be shared upon request to the authors.
