# Supplementary material for: Functional ultrasound (fUS) detects mild cerebral alterations using canonical correlation analysis denoising and dynamic functional connectivity analysis
Source: Imaging Neurosci (Camb). 2025 Sep 2;3:IMAG.a.128. doi: 10.1162/IMAG.a.128 (PMC12406050; doi:10.1162/IMAG.a.128)
Supplement: Supplementary Material [file IMAG.a.128_supp.pdf]

Supplementary 1: seed based maps before/after CCA denoising

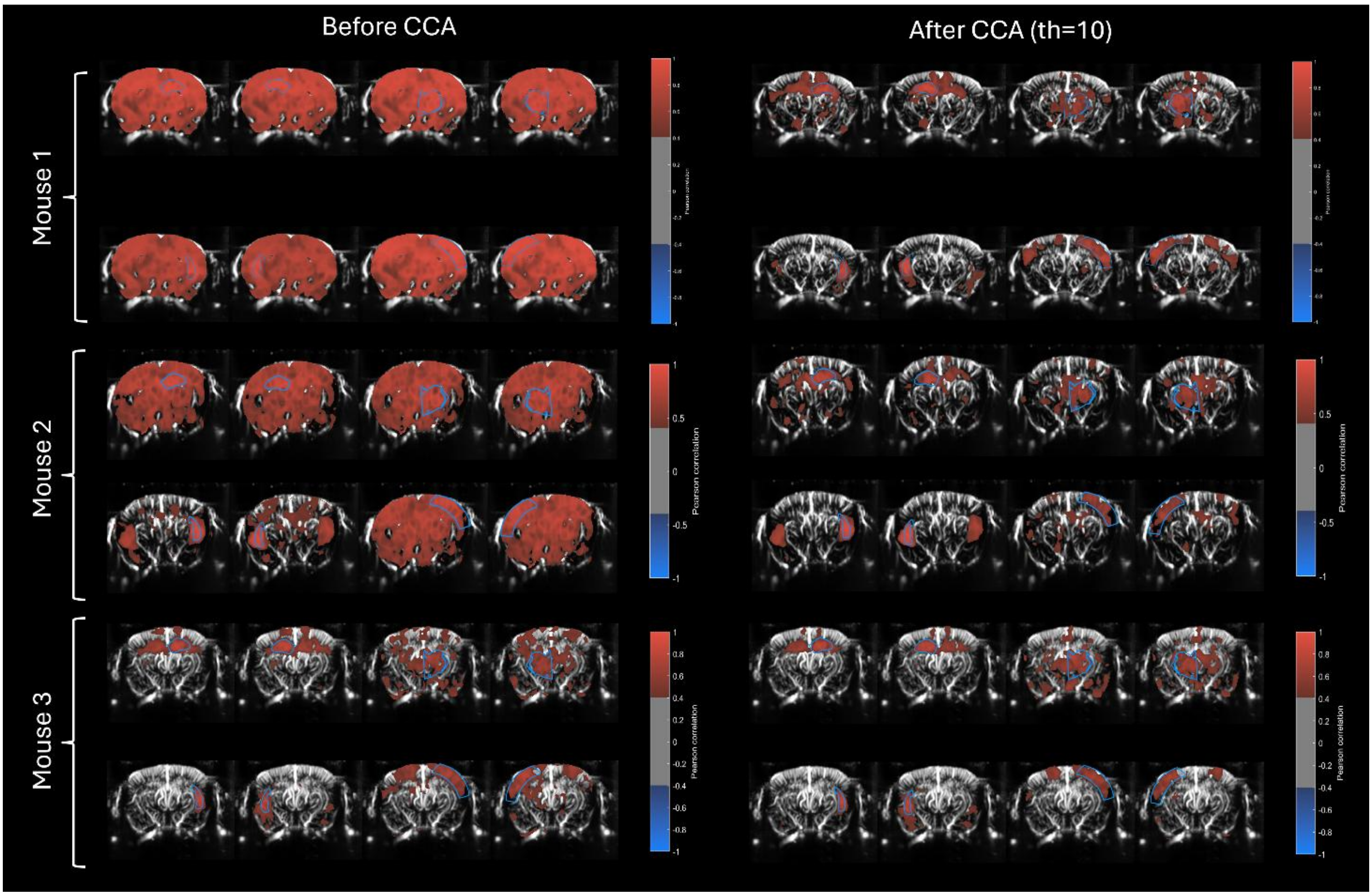

Supplemental figure 1: impact of CCA denoising on seed-based analysis in coronal section in three representative mice, presenting various level of artefacts and noise level. On the top mouse, very strong global correlation before CCA (left) makes any seed correlate largely with the rest of the brain, while after CCA (right) one seed present large correlation values mostly with the contralateral functional area. On the bottom mouse, it can be seen that in the case of very low level of noise, the CCA preprocessing (even if using the same threshold than the other mice) does not impact the signals and leaves the inter-hemispheric connectivity that was present mostly untouched.

Supplementary 2: K-means for K=3 and K=5

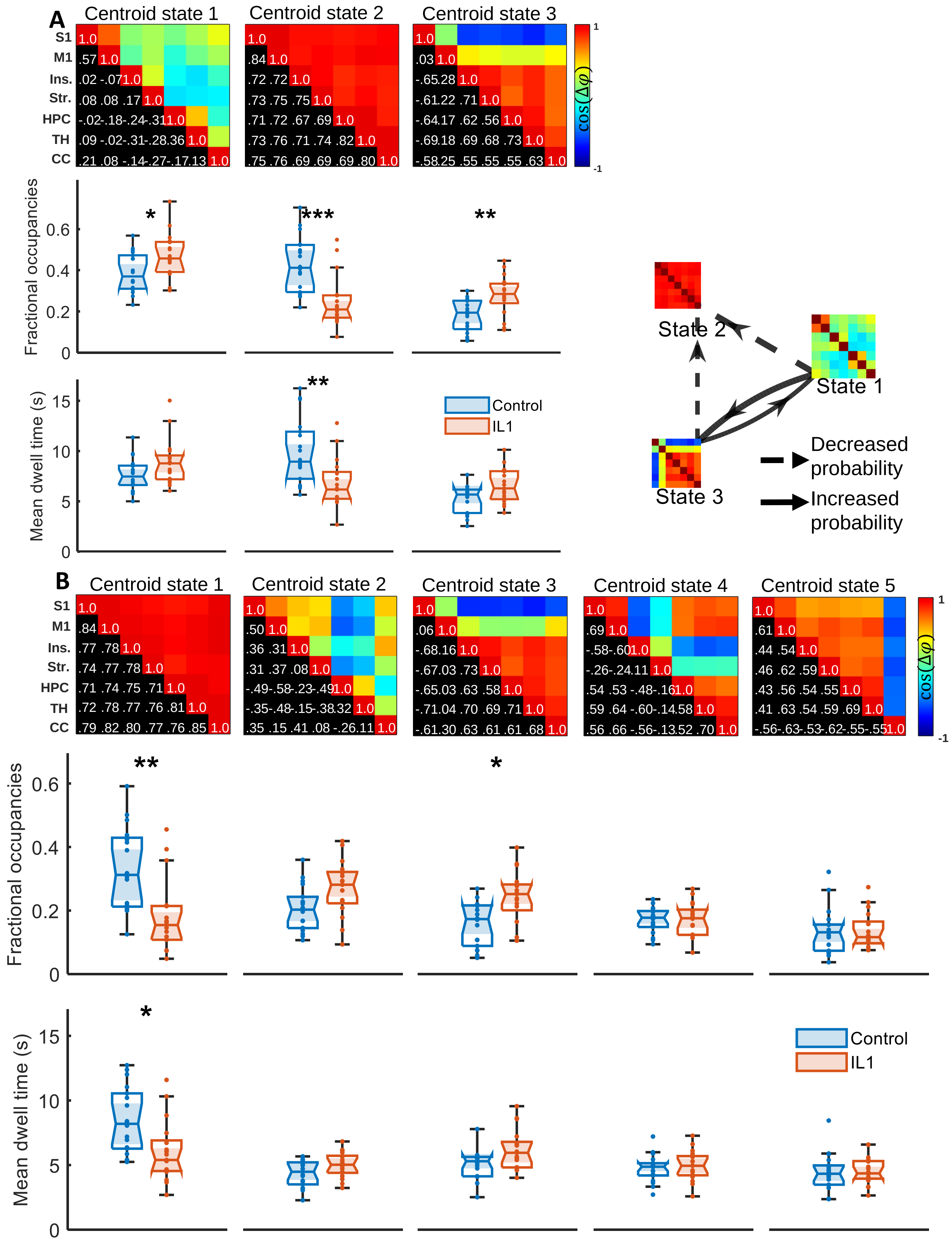

Supplementary Figure 2. Dynamic functional connectivity (dFC) analysis on CCA denoised data with the K-means algorithm show consistent differences between groups for A) K=3 states or B) K= 5 states. The K centroid states are displayed as matrices with their associated fractional occupancies and mean dwell time for each group below**.** For each centroid state, two-way t-test analysis was performed between fractional occupancies of the control group (blue) and the IL-1β group (orange), followed by Bonferroni correction for multiple comparisons with a degree of freedom equals to K–1 (i.e respectively 2 and 4) (* $p < 0.05$, ** $p < 0.01$, *** $p < 0.001$). Mann-Whitney U test was performed between the mean dwell time of both group for each centroid state, followed by Bonferroni correction for multiple comparisons with a degree of freedom equals to K (i.e. respectively 3 and 5)(* $p < 0.05$, ** $p < 0.01$, *** $p < 0.001$). For K=3, the significant differences in states transitions are also displayed as an oriented graph.

Supplementary 3: FC with GSR: brain states and transitions between states

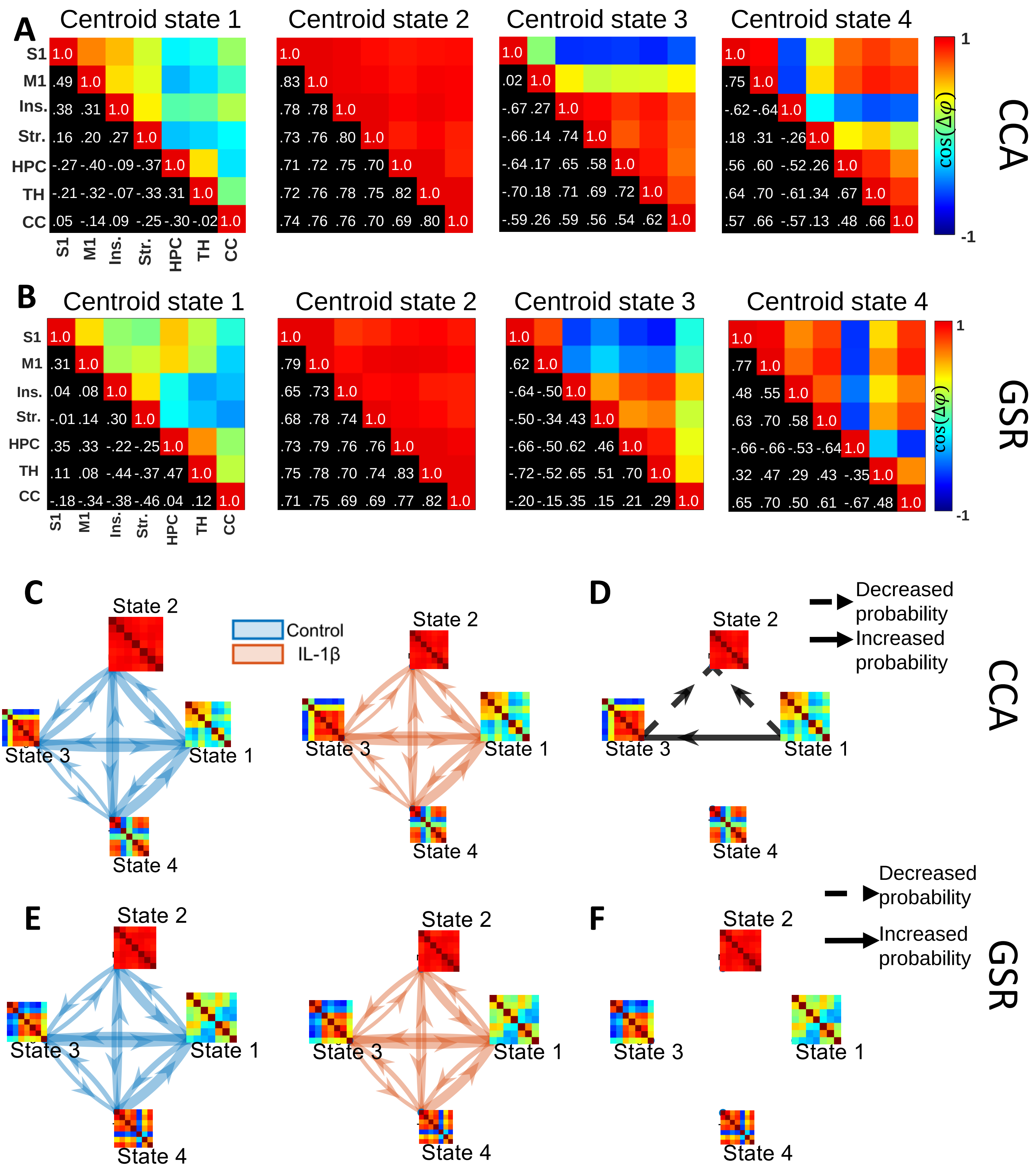

Supplementary Figure 3. A. The centroid states obtained after CCA denoising (as in fig 5) for a side-by-side comparison with the **B.** Centroid states obtained GSR denoising. The states are very similar, except for state 4, which anyway does not participate in the difference between the IL-1-β and the control group. **C & D** same as in figure 5 (CCA) for a side by side comparison with the **E.** States transitions for the control group (blue arrows) and the IL-1β group (orange arrows) after GSR denoising. The width of the arrows is proportional to the probability of transition from one state to another. The size of the matrices is proportional to their fractional occupancies. **F.** Significant differences in state transition for GSR denoising : none**.** Two-way t-test was performed followed by Bonferroni correction for multiple comparisons with a degree of freedom equal to the number of transitions (i.e. 12). Dashed arrows represent a significantly decreased probability of transition, and the full arrow a significantly increased probability of transition ($p<0.05$).

Supplementary 4: effect of changing CCA threshold on FC metrics

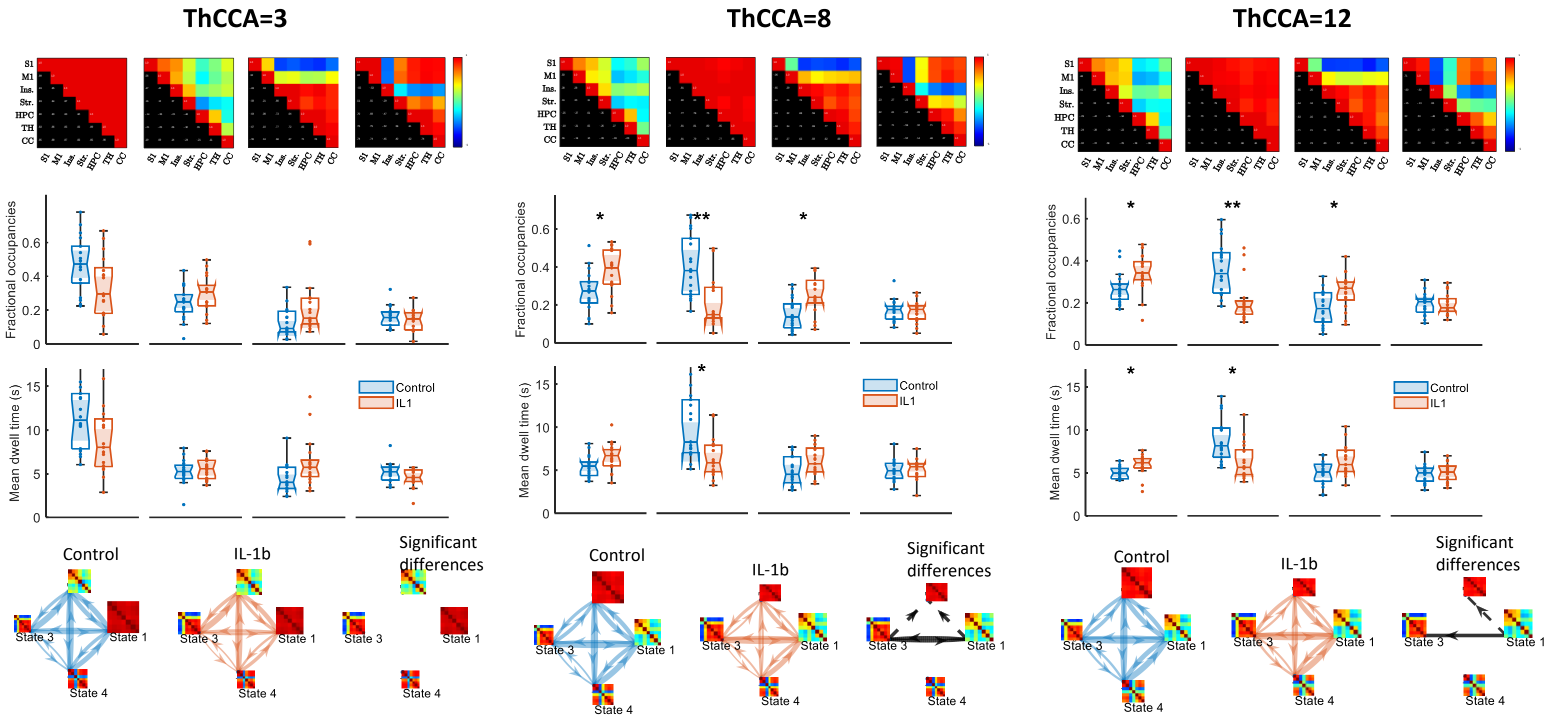

Supplementary Figure 4: Use of a variety of thresholds for CCA denoising and its effect on dynamic functional connectivity metrics quantifications. If the threshold is too low the significant difference between the inflammation group and controls stays concealed, but if the threshold is near (8 or 12) the determined optimum (10, according to our correlation with motion criteria) the conclusions stay the same.
